# Assessing the translation of diagnostic colorectal cancer biomarkers from bench to bedside

**DOI:** 10.3389/fmed.2025.1598697

**Published:** 2025-09-15

**Authors:** Alice E. Baggaley, Jade Kabbani, Katerina Vanessa Savva, Prerana Gogoi, James M. Kinross, Melody Zhifang Ni, Christopher J. Peters

**Affiliations:** ^1^Department of Surgery and Cancer, Imperial College London, London, United Kingdom; ^2^New Cross Hospital, Wolverhampton, United Kingdom

**Keywords:** translational science, colorectal cancer, biomarker, clinical utility, clinical implementation

## Abstract

**Background:**

Colorectal cancer is the second leading cause of cancer mortality worldwide. Evidence demonstrates that screening identifies patients with earlier disease to improve survival. It is imperative that diagnostic biomarkers are efficiently translated to clinical practice.

**Aim:**

We aimed to assess the state of biomarker translation by comparing the number of diagnostic colorectal cancer biomarkers in clinical use (successful) with the total number of discovered diagnostic colorectal cancer biomarkers not yet clinically useful (stalled).

**Method:**

A literature search identified all published diagnostic colorectal cancer biomarkers. Data was extracted from eligible papers including biomarker name, authors, journal and impact factor, and publication date. Guidelines from national and international authorities/groups were searched and a clinical expert consulted to identify successful diagnostic colorectal cancer biomarkers. Outcomes included number of stalled and successful diagnostic colorectal cancer biomarkers discovered in the literature, number of publications for each biomarker and their highest journal impact factor.

**Results:**

After screening >32,000 papers, 2,910 diagnostic colorectal cancer biomarkers were identified. Of these, four were approved for use in patient care at the time of review, representing a translation rate of just 0.14%. Successful biomarkers were found to have a higher publication frequency, and higher journal impact factor when compared to stalled biomarkers.

**Conclusion:**

This study demonstrates a profoundly low rate of translation of diagnostic colorectal cancer biomarkers and identifies the huge translation gap within this field. 84% of biomarkers have only one published paper, suggesting a lack of progression toward approval and commercialisation. There is great scope for improved biomarker translation.

## Introduction

Globally, colorectal cancer (CRC) was the second leading cause of cancer-related deaths in 2022 and remains the third most frequently diagnosed (1). Evidence has demonstrated that earlier CRC diagnosis leads to improved outcomes (2), and National Health Service (NHS) England has set out the aim to diagnose 75% of cancers in their early stage by 2028. Alongside this, the UK government has committed £79 million to the Accelerating Detection of Disease programme (3). Improvement in CRC outcome will be driven by multiple factors but is likely to require development of new, more effective diagnostic/early detection biomarkers.

Very few diagnostic CRC biomarkers are utilised in global screening programmes. In the United Kingdom (UK), the stool faecal immunochemical test (FIT) is the primary tool used in the National Bowel Cancer Screening programme. It is also deployed as a triage test for symptomatic patients (4). Prior to the adoption of FIT, guaiac faecal occult blood testing (gFOBT) was used for screening but was phased out from 2016 after UK National Screening Committee recommendations. In the United States of America however, a larger selection of CRC biomarkers is available to clinicians/patients for screening. These include FIT, but also a more recently developed test combining FIT and stool DNA (Cologuard). There is also the option for patients to choose a blood-based biomarker, methylated Sept9 (Epi proColon).

Despite being used in both the UK and USA bowel cancer screening programmes, FIT suffers from low sensitivity and specificity (especially as a diagnostic tool for pre-cancerous polyps), as well as poor acceptance from patients (5, 6). Limitations of the screening programme are highlighted by the fact that 29% of colon cancer and 10% of rectal cancer cases in the UK are still being diagnosed as an emergency (7). Therefore, there is great interest from academics, clinicians, and industry to search for more effective biomarkers. However, very few biomarkers move from discovery to clinical adoption; Savva et al. describe a translation rate of just 0.94% for breast cancer recurrence biomarkers (8). Biomarkers have traditionally been described as moving sequentially through a ‘pipeline’ from discovery to assay validation, clinical validation, and clinical utility, before being translated. Multiple factors contribute toward blockages in this pipeline (9), but little work has been done to quantify the scale of the issue within specific research fields.

This novel study addresses this gap by quantifying the current state of translation of diagnostic CRC biomarkers, by tracking the progress of these biomarkers toward clinical adoption in the literature. By identifying the true scale of the failure of biomarker translation, we hope to galvanise efforts to improve the current translation process and reduce academic wastage.

We hypothesis that there will be a large translation gap in the number of discovered versus clinically useful CRC biomarkers and our objective is to assess if the number of publications, publication frequency and impact factor of the journal used have any bearing on the translation status. This involves performing a literature search to identify all the diagnostic CRC biomarkers discovered and then compare them with known translated diagnostic CRC biomarkers.

## Materials and methods

### Search strategy and selection criteria

A systematic literature search was conducted in Ovid Medline and Embase databases in August 2023 to identify all publications exploring diagnostic CRC biomarkers. No date limit was set, and keywords such as “colorectal cancer,” “biomarker,” and “diagnosis,” were included (full list of search terms available in Appendix A). Articles included were primary papers (involving humans, cell lines, or animal models) that mentioned CRC, a biomarker, and reference to diagnosis/detection/screening. Exclusion criteria involved secondary research and studies not published in English. Imaging biomarkers were also excluded as these typically aim to enhance existing diagnostic technology and are not stand-alone entities. Studies involving neuroendocrine or gastrointestinal stromal tumours, and biomarkers for diagnosing Lynch syndrome were also excluded as this paper focuses on the primary type of CRC, adenocarcinoma. Commentaries, editorials, conference abstracts and reviews were also excluded, as were any papers not peer reviewed (e.g., papers on preprint service medRxiv).

Articles identified in the literature search were uploaded to the Covidence platform (Veritas Health Innovation, Melbourne, Australia). Duplicates were removed automatically by Covidence, and any that were not detected by the software were manually removed by the reviewers (AEB & JK). Papers were screened by abstract for eligibility by two reviewers (AEB & JK), with conflicts resolved by a third reviewer (KVS).

### Data extraction

Eligible papers were exported to Microsoft Excel for full text review, and relevant papers had their data extracted and manually tabulated. The data extracted from each publication included author, journal, publication date, biomarker/s identified, and journal impact factor. Data from 10% of papers was extracted by three reviewers independently (JK, AEB, and PG) to assess inter-reviewer disparity. For papers that presented multiple biomarkers, these were extracted as individual biomarkers, unless they were tested as a panel, in which case they were considered one biomarker. Biomarkers were checked for multiple spellings or abbreviations so that recurring biomarkers were counted together. The impact factor of each publication journal, at the time of publication, was retrieved from online databases (10). For biomarkers with more than one paper published, the journal with the highest impact factor was recorded and used for analysis.

### Approved diagnostic CRC biomarkers

Successful biomarkers were defined as those approved for use by clinical authorities and guidelines, including the National Institute for Health and Care Excellence (NICE) (4), British Society of Gastroenterology, Association of Coloproctology of Great Britain and Ireland, Public Health England, American Society for Clinical Pathology, College of American Pathologists, Association for Molecular Pathology, the American Food and Drug Administration (FDA) (11, 12), American Society of Clinical Oncology, European Society for Medical Oncology (ESMO), and Asian Pacific Association of Gastroenterology (APAGE) at the time of the literature search. Biomarkers discovered in the wider literature but not used or approved for use in clinical situations were classed as stalled. Guidelines were searched and the list of successful diagnostic CRC biomarkers was confirmed via discussion with a consultant colorectal surgeon (JMK).

### Statistical analysis

Graphs were generated using GraphPad Prism v8.0.0 (GraphPad Software, San Diego, California USA). Data analysis was conducted using Microsoft Excel and IBM SPSS Statistics v28.0 (IBM Corp., Armonk, N.Y., USA). Normality of data for successful and stalled biomarkers was assessed using a Kolmogorov–Smirnov test. Differences between impact factor and average publication frequency of the two groups were assessed for significance using a Mann Whitney U test. Differences between the number of publications for successful biomarkers before and after clinical translation were also evaluated using a Mann Whitney U test. Binary logistic regression was used to assess the probability of a biomarker’s translation status with respect to the variables of publication history and journal impact factor. A *p* value <0.05 denoted significance.

## Results

### Literature search

**Figure 1:**
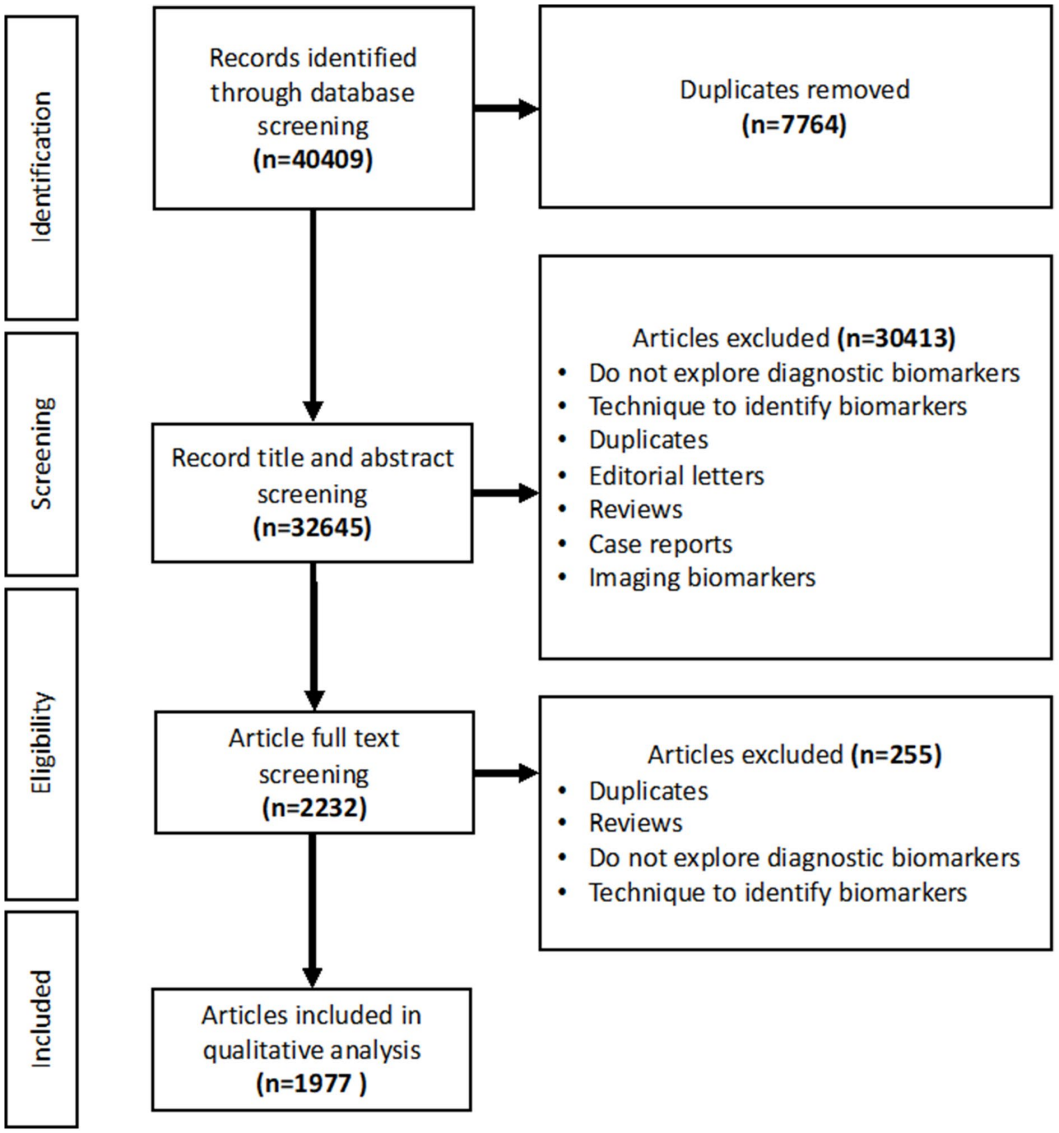
PRISMA flowchart showing the studies included in the title and abstract screening, full text screening and qualitative analysis.

Following the literature search, 40,409 papers were imported, with 7,764 removed as duplicates, providing 32,645 to screen. Abstract and title screening produced 2,232 articles to be included in the full text screen. Following the exclusion of papers that did not fulfil the inclusion criteria, 1,977 papers were included in the final analysis (Figure 1).

### Translation rate for diagnostic colorectal cancer biomarkers

A total of 2,910 diagnostic CRC biomarkers (both individual markers and panels) were extracted from the literature. Guidelines from healthcare authorities and organisations were screened, and a CRC expert consulted to identify successful biomarkers, as detailed in methods and Table 1. Only four diagnostic biomarkers were defined as successful; FIT, gFOBT, mSEPT9, and Cologuard, with the remainder categorised as stalled (*n* = 2,906). This data gives a survival rate for diagnostic CRC biomarkers within the literature of 0.14%. Normality of the data was examined using a Kolmogorov-Smirnov test and found to be non-parametric.

**Table 1:** Diagnostic CRC biomarkers recommended for use as per national guidelines and approval bodies.

Biomarker and commercial name	Biospecimen type	Approval body/guidelines	Year of approval
Guaiac faecal occult blood test (gFOBT)	Stool	NICE, ASCP, CAP, AMP, ASCO, FDA, PHE, ACS	2006 (24)
Faecal immunochemical test (FIT)	Stool		2019 (25)
Methylated Septin9 (mSEPT9) Epi proColon	Blood (plasma)	FDA	2016 (11, 26)
FIT-DNA testing of β-actin/mutant KRAS/BMP3/NDRG4/faecal haemoglobin Cologuard	Stool	FDA	2014 (12)

NICE, National Institute for Health and Care Excellence; ASCP, American Society for Clinical Pathology; CAP, College of American Pathologists; AMP, Association for Molecular Pathology; ASCO, American Society of Clinical Oncology; FDA, American Food and Drug Administration; PHE, Public Health England; ACS, American Cancer Society.

### Publication pattern

**Figure 2:**
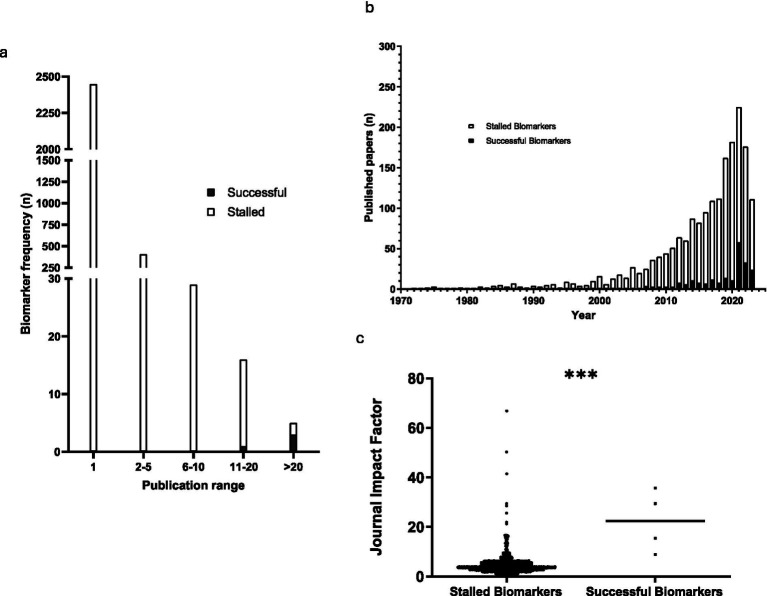
Publications and biomarkers identified from 1972–2023. **(a)** A bar chart showing the frequency of diagnostic CRC biomarkers for publication ranges 1, 2–5, 6–10, 11–20, and >20 (*n* = 2,910). **(b)** A bar chart showing the number of original papers published each year from 1972–2023 (successful *n* = 210, stalled *n* = 1,881). **(c)** A scatter plot showing the highest journal impact factor for successful (*n* = 4) and stalled biomarkers (*n* = 2,906). The horizontal lines denote the median value of each group. *** denotes *p* < 0.01.

The publication frequency for each biomarker is demonstrated in Figure 2a and groups biomarkers into five publication frequency ranges: 1, 2–5, 6–10, 11–20 and >20. The low publication frequency of stalled biomarkers is highlighted by the fact that 2,449 biomarkers (84.3%) had only 1 publication associated with them, 407 (14.0%) had 2–5 publications, 29 (1.0%) had 6–10 publications, 16 (0.6%) had 11–20 publications, and only 5 (0.2%) had >20 publications. In contrast, three of the successful biomarkers had >20 publications, and one had between 11 and 20 publications.

There was a larger number of publications within the stalled group of biomarkers (1881 vs. 210) compared to the successful group (Figure 2b), but this reflects the very large number of biomarkers within that cohort (2906). A number of publications included both successful and stalled biomarkers, and hence the total publication number exceeds the number of papers extracted in the literature search (1977), as some are counted in both cohorts. The successful biomarkers had a high publication frequency per biomarker (average 52.5 publications/biomarker) compared to the stalled group (average 0.6 publications/biomarker).

Since many publications included more than one biomarker, the number of biomarkers is higher than the total number of publications. A Mann-Whitney U test was performed to compare the publication frequency in both groups and was significant (*p* < 0.0001). There has been an exponential increase in diagnostic CRC biomarker research within the timeframe of this study (Figure 2b).

**Figure 3:**
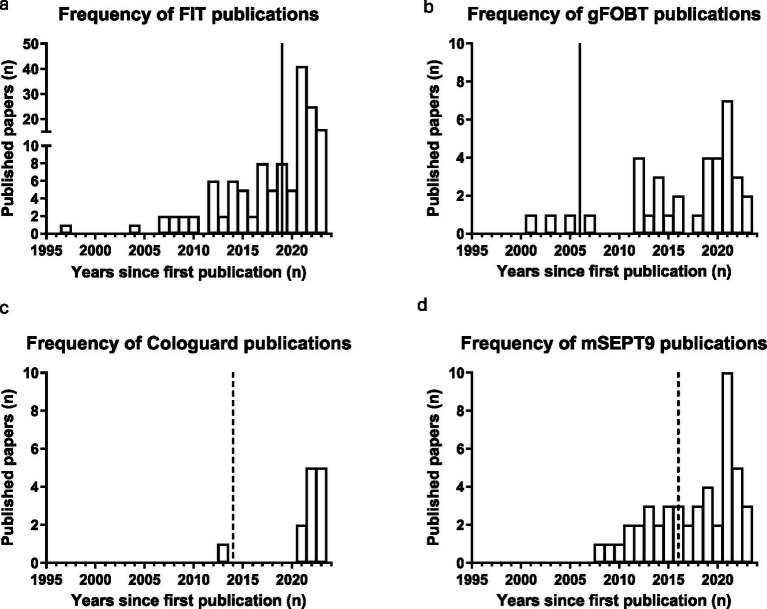
Bar charts demonstrating publication frequency for each successful diagnostic CRC biomarker. **(a)** FIT, **(b)** gFOBT, **(c)** Cologuard, **(d)** mSEPT9. Dashed line: year of FDA approval; straight line: year added to UK Bowel Cancer Screening Programme.

To further investigate publication history, bar charts demonstrating the publication frequency of the four successful biomarkers (FIT, gFOBT, mSEPT9, and Cologuard) were plotted (Figure 3).

The year successful biomarkers were approved for use by healthcare authorities (Table 1) was used to investigate potential correlations between the number of publications and biomarker approval. There was no significant increase in the average number of publications per year once a biomarker was approved for clinical use (*p* = 0.3429, Mann Whitney U).

**Figure 4:**
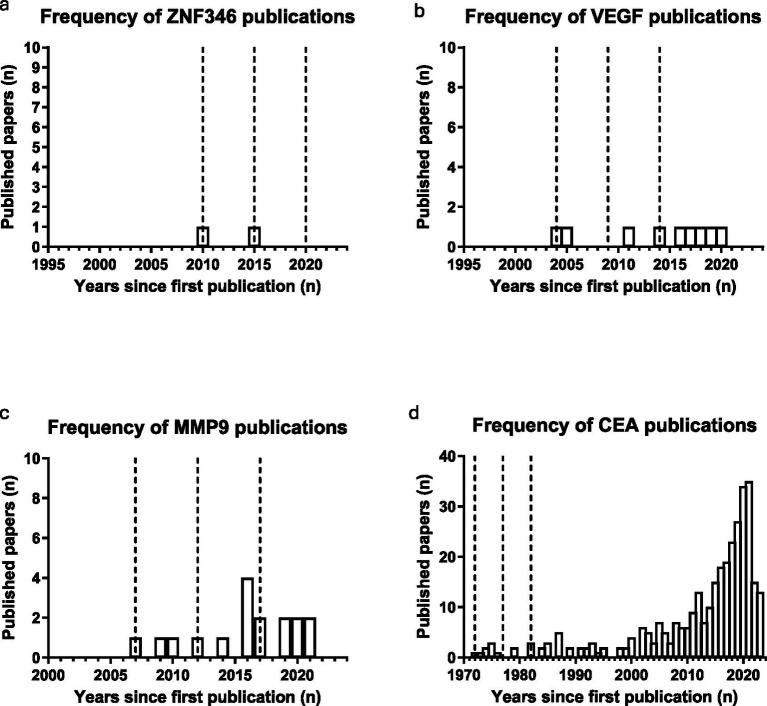
Bar charts demonstrating publication frequency for one stalled biomarker from each publication frequency range. **(a)** 2–5: ZNF346, **(b)** 6–10: VEGF, **(c)** 11–20: MMP9, **(d)** >20: CEA. Dashed lines represent 5-year intervals from first publication.

Average publication history for the successful group of biomarkers was calculated to be 19 years (FIT = 27, gFOBT = 23, mSEPT9 = 16, Cologuard = 11). Due to the large disparity in size of the successful and stalled group of biomarkers (successful biomarker group: 4 vs. stalled biomarker group: 2906), the average biomarker publication history was measured for a random selection of five biomarkers within each publication frequency range (2–5, 6–10, 11–20, >20). Successful biomarkers had a higher average publication history than biomarkers within the 2–5 range (8 years; ZN346, glutamine, mir-210, mir-101 and transferrin), and the 6–10 frequency range (15.8 years; VEGF, AFP, CRP, MMP7 and mir-29a). However, the randomly selected stalled biomarkers within the 11–20 frequency range (p53 antibodies, calprotectin, SDC2, CA125, and MMP9) had a higher average publication history (23.4 years), as did stalled biomarkers within the >20 publications category (31.8 years; CEA, CA19-9, CEA/CA19-9 panel, M2PK and mir-21). The publication history for one random biomarker from each frequency range is displayed in Figure 4. Stalled biomarkers have sparser distribution over time when compared to successful biomarkers, as indicated by Figure 4. Binary logistic regression was used to model the impact of publication frequency on the probability of a biomarker being successfully translated. Publication frequency was found to be a significant factor, with a *p* value of 0.0047.

### Journal impact factor

The maximum impact factor for publications for the successful and stalled biomarkers were recorded and compared (Figure 2c). For biomarkers with >1 publication, the highest impact factor recorded was used. The minimum, mean, and maximum impact factor for stalled biomarkers was 0.00, 4.467, and 66.85 respectively, whereas for successful biomarkers it was 8.9, 22.37, and 35.70. Successful biomarkers were found to be published in journals with significantly higher median impact factor than stalled biomarkers (*p* < 0.0001, Mann Whitney U test). Binary logistic regression was used to model the importance of the journal impact factor on the probability of a biomarker being successfully translated. Impact factor was found to be a significant feature (*p* = 0.0011).

## Discussion

The key outcomes from this study are (i) the quantification of the scale of the translation gap for diagnostic CRC biomarkers, with only 0.14% of those found in the literature being used by patients, and (ii) that successful biomarkers are associated with a significantly higher frequency of publications and published in higher impact journals. This strikingly low rate of translation speaks to the complex issues and barriers along the biomarker translational pipeline (13). Previous work by our group has demonstrated a similar translation gap within the field of prognostic breast cancer biomarkers (8).

The strength of this study lies in its unbiased approach to reviewing all discovered diagnostic CRC biomarkers in the literature. It is the first time that the actual number of biomarkers within this research field has been elucidated and offers a stark demonstration of the inefficiency of the current translation process. It is a simple study that proves our hypothesis and provides a compelling argument for improving biomarker translation efficiency. Previous studies have looked more broadly into discovered candidate proteins as cancer biomarkers, but not specifically within the field of diagnostic CRC biomarkers (14, 15).

In terms of limitations, is likely that this study is an underrepresentation of the total number of papers and biomarkers involved in this field, as later work involving individual biomarker systematic searches (data not shown) resulted in higher numbers of eligible papers. By widening the net to include all possible diagnostic CRC biomarkers, the ability to identify every paper for each biomarker was diminished. The large discrepancy in size between the two groups also made statistical analysis more limited. To confirm the robustness of our findings despite imbalanced group sizes, permutation tests were done to compare groups using Python 3.11. There was a statistically significant difference in publication frequency between successful and stalled biomarkers, with a difference of 55.43 publications (95% CI: −0.64 to 2.61; *p* = 0.0014) per biomarker. Similarly, the maximum journal impact factor per biomarker also showed a significant difference between groups, with an observed difference of 16.73 (95% CI: −2.47 to 3.98; *p* = 0.0018). These results remain consistent with the primary analysis, supporting the reliability of our conclusions.

Although our analysis did not directly assess the sensitivity or specificity of individual biomarkers, these diagnostic performance metrics are known to play a critical role in clinical adoption. The absence or inconsistency of such data across studies represents a significant translational barrier and should be prioritised in future evaluations.

Given the volume of included studies, it was beyond the scope of this review to examine additional factors, such as diagnostic performance, assay platform, or biomarker type, for successful versus stalled biomarkers. However, our group has previously demonstrated no significant differences in performance metrics (e.g., sensitivity, specificity, AUROC, positive predictive value) between successful and stalled prognostic biomarkers in breast and colorectal cancer (16). It is likely that most published biomarkers exhibit a minimum acceptable level of diagnostic performance due to publication bias toward positive findings (17), and that other factors are primarily responsible for hindering translation.

Future work should explore how diagnostic performance metrics interact with practical assay features, such as cost-effectiveness, automation potential, and time-to-result—to influence translational success. A more comprehensive understanding of these interactions could support more effective biomarker development and prioritisation, as exemplified by recent efforts to build structured decision-support tools like the Biomarker Toolkit (16).

Despite the exponential increase in diagnostic CRC biomarkers published papers, only four are currently approved for routine use. Most of the stalled biomarkers in this study (84.3%) have only ever featured in one published paper. Only five stalled biomarkers are associated with >20 publications. Two of those biomarkers (CEA, CA19-9) are biomarkers that are in routine use for other indications (CRC recurrence, pancreatic cancer monitoring), but not approved for use in diagnosing CRC. However, due to the dearth of approved diagnostic CRC biomarkers, these were often used in papers as the reference standard, which may go some way to explain their high publication frequency in this study, despite their stalled status.

Three of the successful biomarkers had >20 papers associated with them, and the other one was within the 11–20 publication range. Overall, the average publication history for the successful group was higher than the randomly selected stalled biomarkers within the lower publication frequency ranges (2–10), but not the higher ranges (11–20, >20). This reflects that using publication history alone is a poor indicator of future success, and may indicate repeated discovery/validation studies, without meaningful progression along the biomarker development pipeline. It also suggests that it is not for lack of time that many stalled biomarkers have not been adopted. Successful biomarkers are associated with publications of significantly higher median impact factor, but the range within the stalled group is large (0–66.85), indicating again that impact factor alone is a poor marker of future success. Indeed the use of journal impact factor is widely acknowledged to be a blunt and inaccurate measure of a paper’s quality, and there has been a drive to do away with this metric entirely (18).

The high rate of biomarkers associated with only one paper highlights a potential flaw in academia, where it remains easier to publish discovery studies, which may help maintain scientists’ grants and career progression, whilst well-powered validation studies and cost-effectiveness studies are less likely to be performed (15). Academics often lack the requisite skill set for these clinical utility studies, and lack of transparency about what is required from the regulatory/commissioning bodies may also hinder the path to translation (9). This sets up a loop of continual biomarker discovery, without ‘less glamourous’ studies such as external reproducibility or decisional analysis being undertaken. There is an urgent need for larger biomarker consortia to be established to combine skill sets and stakeholders to drive candidates through to clinical adoption. The UK for instance has fourteen HealthTech Research Centres sponsored by the National Institute of Health Research, that have been set up to help the translation of early technology and biomarkers.

This review covered more than 30 years, providing representative information for translation, as more recent studies have corroborated the limited translation of CRC biomarkers (19, 20). Since 2022, two new non-invasive biomarkers for colorectal cancer have been approved, including the FDA-approved blood-based test Shield and the stool-based RNA test ColoSense (21, 22). Despite employing a systematic and comprehensive search strategy, it is possible that some relevant biomarkers or publications were not captured. This may be due to inherent limitations such as publication bias, inconsistent terminology used across studies, or language restrictions. While this may have affected the absolute number of biomarkers identified, it is unlikely to impact the overall conclusion regarding the low rate of clinical translation. If anything, the omission of additional biomarkers would likely widen the translational gap observed in this study.

Each stalled biomarker in the literature represents academic wastage (23). Several factors contribute to the stagnation of biomarkers in the translational pipeline, including lack of robust multicentre validation studies, technological barriers such as low reproducibility or complex assay requirements, and challenges in meeting regulatory and reimbursement criteria. Additionally, limited collaboration between academic researchers and industry partners further impedes successful translation. This study reinforces the need for alternative/supplementary methods of identifying the best biomarker candidates to take forward. One proposed solution to this bottleneck is the application of the Biomarker Toolkit, developed by our group (16). The Toolkit comprises a series of checklist attributes that can be used to score biomarker research across several domains: Analytical Validity, Clinical Validity, and Clinical Utility. This has been developed to assess biomarker potential and then, more importantly, guide their further development by recommending future studies/identifying research gaps. Future work looks to apply the Biomarker Toolkit to these diagnostic colorectal cancer biomarkers to further validate the Toolkit, but also to highlight which biomarkers have the greatest potential to be clinically adopted. Those with the highest score could be taken up by research groups with early industry collaboration to streamline their translation and adoption. This study highlights the current state of translation for diagnostic colorectal cancer biomarkers and the need for improved clinical adoption.

## Data Availability

The original contributions presented in the study are included in the article/Supplementary material, further inquiries can be directed to the corresponding author.
