# Supplementary material for: Assessing the translation of diagnostic colorectal cancer biomarkers from bench to bedside
Source: Front Med (Lausanne). 2025 Sep 15;12:1598697. doi: 10.3389/fmed.2025.1598697 (PMC12477240; doi:10.3389/fmed.2025.1598697)
Supplement: Supplementary file 1 [file Supplementary_file_1.docx]

| **Supplementary Appendix A**: Search terms to retrieve Colorectal cancer biomarkers | | | |
| --- | --- | --- | --- |
|  | **EMBASE** |  | **MEDLINE** |
| 1 | Biomarkers, Tumor/ | 1 | Biomarkers, Tumor/ |
| 2 | Biomarker*.mp. or Biomarkers, Tumor/ | 2 | Biomarker*.mp. or Biomarkers, Tumor/ |
| 3 | Bio-marker*.mp. | 3 | Bio-marker*.mp. |
| 4 | Marker* biological.mp. | 4 | Bio* marker*.mp. |
| 5 | Molecular marker*.mp. | 5 | Marker* biological.mp. |
| 6 | Signature molecule*.mp. | 6 | Molecular marker*.mp. |
| 7 | Pathology marker*.mp. | 7 | Signature molecule*.mp. |
| 8 | Blood marker*.mp. | 8 | Pathology marker*.mp |
| 9 | Feces/ or F?eces marker*.mp. | 9 | Blood marker*.mp. 2097 |
| 10 | Feces/ or F?eces marker*.mp. | 10 | Feces/ or F?eces marker*.mp. |
| 11 | F?ecal marker*.mp. | 11 | Feces/ or F?eces marker*.mp. |
| 12 | Urin* marker*.mp. | 12 | F?ecal marker*.mp. |
| 13 | Blood marker*.mp. | 13 | Urin* marker*.mp. |
| 14 | Stool marker*.mp. | 14 | Blood marker*.mp |
| 15 | Diagnos*.mp. | 15 | Stool marker*.mp. |
| 16 | Screen*.mp. | 16 | Diagnos*.mp. |
| 17 | Detect*.mp. | 17 | Screen*.mp. |
| 18 | "Early Detection of Cancer"/ or Early cancer.mp. | 18 | Detect*.mp. |
| 19 | Recogni*.mp. | 19 | "Early Detection of Cancer"/ or Early cancer.mp. |
| 20 | Cancer*.mp. | 20 | Recogni*.mp. |
| 21 | Neoplasm*.mp. | 21 | Cancer*.mp. |
| 22 | Malignan*.mp. | 22 | Neoplasm*.mp. |
| 23 | Tumo?r*.mp. | 23 | Malignan*.mp. |
| 24 | Malignant neoplasm*.mp. | 24 | Tumo?r*.mp. |
| 25 | Malignant tumo?r*.mp. | 25 | Malignant neoplasm*.mp. |
| 26 | Carcinoma/ or Carcin*.mp. | 26 | Malignant tumo?r*.mp. |
| 27 | Approv*.mp. | 27 | Carcinoma/ or Carcin*.mp. |
| 28 | Accepted.mp. | 28 | Approv*.mp. |
| 29 | Official.mp. | 29 | Accepted.mp. |
| 30 | Guideline*.mp. | 30 | Official.mp. |
| 31 | NICE.mp. | 31 | Guideline*.mp. |
| 32 | FDA.mp. | 32 | NICE.mp. |
| 33 | translated.mp. | 33 | FDA.mp. |
| 34 | 1 or 2 or 3 or 4 or 5 or 6 or 7 or 8 or 9 or 10 or 11 or 12 or 13 or 14 or 15 | 34 | translated.mp. |
| 35 | 16 or 17 or 18 or 19 or 20 | 35 | 1 or 2 or 3 or 4 or 5 or 6 or 7 or 8 or 9 or 10 or 11 or 12 or 13 or 14 or 15 |
| 36 | 21 or 22 or 23 or 24 or 25 or 26 or 27 | 36 | 16 or 17 or 18 or 19 or 20 |
| 37 | 28 or 29 or 30 or 31 or 32 or 33 or 34 | 37 | 21 or 22 or 23 or 24 or 25 or 26 or 27 |
| 38 | 35 and 36 and 37 and 38 | 38 | 28 or 29 or 30 or 31 or 32 or 33 or 34 |
| 39 |  | 39 | 35 and 36 and 37 and 38 |
